# Rurality and Outcomes of Patients Undergoing Mechanical Thrombectomy for Acute Ischemic Stroke

**DOI:** 10.1155/ccrp/4995600

**Published:** 2025-01-30

**Authors:** Cassidy Lavin, Jacob Epstein, Alvin Huanwen Chen, Minahil Cheema, Jerry Yang, Alexa Aquino, Angie Chan, Nancy Le, Gillian Cooper, Ambra Palushi, Chad Schrier, Dheeraj Gandhi, Seemant Chaturvedi, Jessica Downing, Quincy K. Tran

**Affiliations:** ^1^University of Maryland School of Medicine, Baltimore, Maryland, USA; ^2^Research Associate Program in Emergency Medicine and Critical Care, Department of Emergency Medicine, University of Maryland School of Medicine, Baltimore, Maryland, USA; ^3^Department of Radiology, Division of Interventional Neuroradiology, University of Maryland Medical Center, Baltimore, Maryland, USA; ^4^National Institute of Neurological Disorders and Stroke, National Institutes of Health, Bethesda, Maryland, USA; ^5^Program in Trauma, R Adams Cowley Shock Trauma Center, University of Maryland School of Medicine, Baltimore, Maryland, USA; ^6^Department of Neurology, University of Maryland School of Medicine, Baltimore, Maryland, USA; ^7^Department of Emergency Medicine, University of Maryland School of Medicine, Baltimore, Maryland, USA

**Keywords:** health services accessibility, ischemic stroke, thrombectomy

## Abstract

**Objective:** To investigate differences in outcomes among patients with acute ischemic stroke from large vessel occlusion (AIS-LVO) transferred from rural and urban hospitals to University of Maryland Medical Center (UMMC) for mechanical thrombectomy (MT).

**Methods:** We identified patients with AIS-LVO transferred to UMMC for MT from July 2016 to June 2023. Primary outcome was good neurologic outcome, defined as 90-day modified Rankin score 0–2. Multivariable logistic regression was used to identify predictors for the primary outcome.

**Results:** We analyzed 526 patients, 233 (44%) transferred from rural hospitals in Maryland. Median NIHSS was 17 [IQR 14–20] and was similar between groups. Patients from state-designated rural hospitals were transferred from a longer distance (difference of 57.8 km, *p*=0.001), but had shorter intervals from last known well time to recanalization (difference 19 min, *p*=0.24). They had similar odds of good neurologic outcome (OR 0.88, 95% CI 0.43–1.78, *p*=0.72).

**Conclusions:** Patients transferred from rural areas for MT for AIS-LVO, at our institution, had a similar likelihood of achieving 90-day mRS 0–2 as those transferred from urban areas.

## 1. Introduction

Stroke is a leading cause of disability and death in the United States [1, 2] and poses a significant cost burden to the American healthcare system [3]. A stroke occurs when blood flow to an area of the brain is decreased by a clot or sudden bleeding. When blood flow to the brain is blocked by a clot, this is called an ischemic stroke, which is the cause of a majority of stroke cases. When a patient presents to the hospital and is diagnosed with an ischemic stroke, they are evaluated for candidacy of intravenous (IV) thrombolytic (clot-busting) therapy and for eligibility for physical removal of the clot, a procedure otherwise known as mechanical thrombectomy (MT) [4]. In recent years, MT has become the standard of care for patients with ischemic stroke caused by large vessel occlusion (AIS-LVO) [5]. This minimally invasive procedure is performed by a specialized Neuro Interventional Radiologist, a position which is most often staffed by comprehensive stroke centers in tertiary and quaternary care hospitals.

Despite advancements in stroke care, significant disparities exist in outcomes between rural and urban patients with AIS [6]. Previous studies have shown that patients treated for stroke in rural areas are more likely to face poorer outcomes compared to their urban counterparts. This phenomenon has previously been attributed to factors such as limited access to specialized stroke centers, longer transportation times, and delays in receiving acute therapies [7]. Studies have shown that rural patients often present with more severe strokes (higher baseline NIH stroke scale scores) and receive acute treatments like IV thrombolysis and endovascular therapy less promptly [6, 8].

A systematic review in 2019 by Dwyer et al. concluded that there is convincing evidence suggesting that rural patients receive less acute stroke care when compared to their urban counterparts [9]. However, the available data had not been used to investigate rural-urban differences in patient outcomes [9]. A study conducted by Shen et al. in 2022 attempted to address this gap in the literature and found no significant difference in functional neurologic outcome between patients living in rural versus urban areas who underwent MT [7]. However, this study was limited to one institution in the Southeastern U.S., noted to be easily accessible by rural patients [7]. Another limitation for the study by Shen et al. was that there was a small sample size, especially the number of rural patients. Investigators were thus unable to account for the barriers of proximity and geography that many rural patients face in other areas of the country, which impact access to appropriate stroke care.

Research examining the discrepancies in access to stroke care, clinical characteristics, hospital courses, and potential complications of rural versus urban patients undergoing MT is limited, making it difficult to fully understand the disparities in care and outcomes for this high-risk group. Understanding these differences can help identify areas where stroke care delivery can be improved to optimize patient outcomes across diverse geographical settings. This study aims to fill this knowledge gap by utilizing a larger sample size of patients who underwent rural and urban transfer to investigate the differences in predictors and hospital courses among patients with AIS-LVO transferred to a quaternary care center for MT from both rural and urban hospitals.

## 2. Methods

### 2.1. Study Setting and Design

Adult patients who sustained IS-LVO and were transferred from a referring hospital for MT at the University of Maryland Medical Center (UMMC) between July 2016 and June 2023 were eligible. Patients were identified retrospectively from our institution's prospectively collected stroke registry, which is maintained by the Stroke Neurology team as part of our standard patient care. UMMC is a Comprehensive Stroke Center (CSC) with an in-house Stroke Neurology team 24 h per day, 7 days per week. The consult team is composed of board-certified vascular attending neurologists, a stroke fellow physician, and a nurse coordinator. When a patient is diagnosed with ischemic stroke at our institution or any referring hospitals without MT capability, the Stroke Neurology team is consulted via phone and determines whether the patient is eligible for MT. Patients who are eligible for MT are emergently transferred to an Intensive Care Unit (ICU) at UMMC for immediate evaluation by the Stroke Neurology and the Neurology Interventional Radiology teams. Patients are subsequently transferred to the Neuro Interventional Radiology suite for MT if appropriate. Following MT, patients are admitted to the Neuro Critical Care Unit (NCCU) for post-thrombectomy care per standard of care. The study was exempted for formal consent, due to its observational nature, by our Institutional Review Board.

### 2.2. Patient Selection

Consecutive patients ≥ 18 years of age who were transferred from an outside facility to our CSC during the study period and underwent MT were included. We excluded patients who did not undergo MT or whose records were insufficient. We also excluded patients with occlusion involving the posterior circulation (basilar arteries), as prior evidence has suggested patients with posterior strokes face different outcomes than those with anterior-circulation strokes [10]. Additionally, we excluded patients who first presented to our institution's Emergency Department or who developed acute stroke while admitted at our medical center, as they did not undergo transfer.

### 2.3. Data Collection

We collected data from our institutional electronic medical records (EMR; https://www.epic.com, Wisconsin, United States), the Neurology Interventional Radiology team and the Stroke Registry. Data extraction was performed according to best practices as described previously [11]. Prior to extracting data, researchers, who were not blinded to the study's hypothesis, were trained by senior investigators using sets of 5 patients' charts. Once accuracy between researchers reached 90%, two research team members reviewed each patients' charts; discrepancies were adjudicated by discussion between the two research team members. The outcome of the data was reported as consensus between the researchers so inter-rater reliability was not measured.

**Figure 1:**
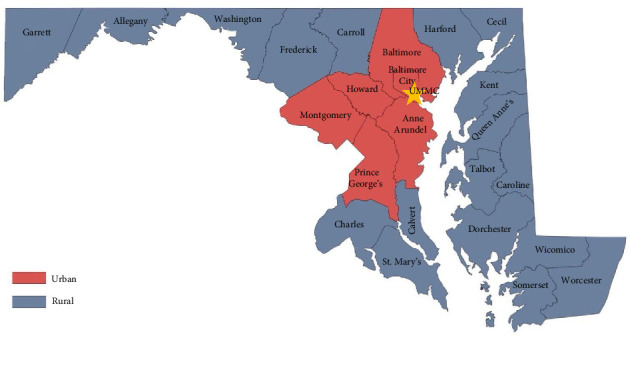
Map of Maryland, representing counties classified as rural versus urban based on state designation [12]. The location of UMMC is represented by a yellow star. Adapted from the Maryland rural council [13].

Data were collected into a standardized password-protected Microsoft Excel spreadsheet (Microsoft Corp, Redmond, WA). Referring centers were identified from review of the EMR; they were classified as rural versus urban centers based on state designation (Figure 1) [12]. Most of the demographic information (age, gender, race, stroke severity (National Institute of Health Stroke Scale [NIHSS], intervals from last known well time to recanalization [LKW-Recan]), interventions (thrombolytic administration, mechanical ventilation, number of passes for recanalization) and outcomes (Thrombolysis in Cerebral Infarction score 2b/2c/3 [TICI 2b/2c/3], intracranial hemorrhage [ICH], subarachnoid hemorrhage [SAH], or hemorrhagic conversion, disposition) were collected prospectively by the Stroke Neurology team for the Stroke Registry. 90 day modified Rankin Scale (mRS) was determined by the Stroke Coordinator, a registered nurse with specialized training in stroke care, either via clinic follow-up visits or by phone call. Additional details regarding patients' hospital course and complications (for example, length of invasive mechanical ventilation, tracheostomy, diagnosis of pneumonia, antibiotics, hospital length of stay [HLOS]) was collected via our EMR. Missing data was imputed using the mean of the population for any given variable, with the exception of 90 day mRS, which was imputed as > 3 if missing. Because most of the relevant data is collected as part of routine clinical evaluation of stroke patients, we did not anticipate lots of missing data.

### 2.4. Outcome Measures

The primary outcome was the rate of good functional independence at 90 day after the onset of stroke symptoms. Good functional independence was defined by the mRS score 0–2. Secondary outcomes included HLOS, and rates of tracheostomy and pneumonia.

### 2.5. Data Analysis

We used descriptive statistics to present the demographic information of our patients. Prior to analysis, data's pattern of distributions was evaluated by histograms. Mean values ( ± standard deviation, SD) or median (interquartile range, IQR) were used for continuous variables, while percentages (%) were used for categorical variables. When appropriate, Student's *t*-test or Mann-Whitney *U* test was used for continuous data, while Fisher's exact test was used to compare categorical data. For comparison, the differences between the groups (effect size) and their 95% Confidence Intervals (95% CI) were also calculated.

We used multivariable logistic regressions to identify variables associated with the dichotomous outcome of functional independence, as defined by 90 day mRS score 0–2. Variables included in the models were selected a priori (Appendix 1). The goodness-of-fit of the data and the collinearity of the variables were assessed by the Hosmer–Lemeshow test and the Variance Inflation Factor (VIF), respectively. We further assessed the performance of the model by using the area under the receiver operating curve (AUROC), which assesses the discriminatory capability of a given model for a dichotomous outcome.

Descriptive statistical analysis was performed in Python, version 3.10.12. The multivariable logistic regressions, goodness-of-fit and AUROC for the multivariable logistic regression were performed with Minitab version 4.0 (https://minitab.com, State College, Pennsylvania, USA). All *p* values < 0.05 were considered statistically significant.

## 3. Results

### 3.1. Patients' Characteristics

**Figure 2:**
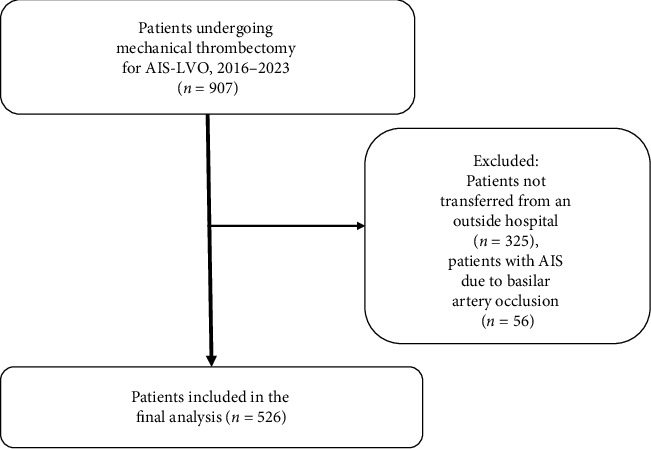
Flow diagram for patient selection.

Our registry identified a total of 907 patients who underwent MT for AIS-LVO; after excluding patients admitted through our Emergency Department or who developed AIS-LVO while already admitted to our hospital, as well as those with AIS due to basilar artery occlusion, we included 526 patients in our analysis (Figure 2), 293 (56%) from urban referring centers while 233 (44%) came from rural hospitals.  Table 1 presented the information between both groups. Patients from rural areas were less likely to have past medical history of diabetes mellitus (14% vs. 22%, difference 8%, 95% CI 0.02–0.15, *p*=0.01) and hypertension (31% vs. 43%, difference 12%, 95% CI 0.030–0.3, *p*=0.01) than were patients transferred from urban hospitals; other baseline characteristics were similar between groups (Table 1). Patients from rural areas were less likely to receive IV thrombolytics (alteplase [tPA] or tenecteplase [TNK]) (25% vs. 34%, difference 9%, 95% CI 0.01–0.17, *p*=0.02).

Patients from rural areas were transferred over a greater ground distance (median 75 [IQR 64–94] km) versus 17 [13–28] km, difference −57.8 km, 95% CI −57.8 to −49, *p* < 0.001).The median time interval from last known well time to recanalization (LKW-Recan) was 441 [321–864] minutes for the population, 431 [330–776] minutes for patients transferred from rural centers, and 450 [315–930] minutes patients transferred from urban centers. Although there was a difference of 19 min, the difference was not statistically significant (95% CI −50 to 1110, *p*=0.24).

### 3.2. Primary Outcome: Good Neurologic Outcome at 90 days

Overall, 106 (20%) patients achieved a 90 day mRS 0–2 (Table 1). Forty-five (45, 19%) patients who were transferred from rural areas achieved 90-day mRS 0–2, compared to 45 (15%) patients from urban areas.

In the multivariable logistic regression (Appendix 2), patients from rural areas had similar odds of achieving 90-day mRS 0–2 as patients from urban areas (OR 0.88, 95% CI 0.43–1.78, *p*=0.72; Table 2).

We found that each additional minute from the time of LKW to recanalization was associated with 1% lower odds of achieving 90-day mRS 0–2 (OR 0.99, 95% CI 0.99–1.00, *p*=0.045). Similarly, higher age in years (OR 0.95, 95% CI 0.93–0.97, *p*=0.001), and higher NIHSS at arrival at our institution (OR 0.92, 95% CI 0.87–0.96, *p*=0.002) was also associated with lower odds of achieving 90 day mRS 0–2.

The *p* value for the Hosmer–Lemeshow test was 0.39, suggesting good fit of the data, and the model demonstrated good discriminatory capability (AUROC = 0.84; Table 2).

### 3.3. Secondary Outcomes: HLOS, Hospital Complications

In our univariate analysis, patients from rural areas had similar rates of developing pneumonia (Table 2), hemorrhagic conversion of ischemic stroke, and of tracheostomy. The median HLOS for patients from rural (6.2 [4.1–13] days) and urban areas (7.9 [4.7–12.9], *p*=0.21) were similar (Table 2).

## 4. Discussion

This study found no significant difference in 90-day neurologic outcomes between patients transferred from rural versus urban hospitals and Emergency Departments to a CSC in Maryland for MT for AIS-LVO. While rural patients were transferred over longer distances, this did not translate to a difference in outcomes. Previous studies suggested that disparities of stroke care existed among patients who were treated at rural versus urban hospitals [6–8]. However, these studies have included all patients with AIS, rather than only those considered candidates for MT, and found that a lower rate of interventions (including thrombolytics and MT) was a main factor driving poorer outcomes among rural patients [6]. Our study agreed with previous observation that rural patients were less likely to receive IV thrombolytics, but all patients ultimately underwent MT, which may explain the disparity between our and prior findings [6]. There was no difference between groups in our univariate analysis. While this nonstatistically significant difference could be due to the fact that our study was underpowered (calculations suggested sample size had a power of 74.4% to detect a 2% difference in 90-day mRS between groups), the nonstatistical difference could be true, since it was supported by our multivariable logistic regression after taking into account many other clinical factors.

Despite a longer transfer distance, we did not identify a significant difference in time between LKW and recanalization via MT between rural and urban patients. Prior studies have suggested that longer intervals from LKW to reperfusion (either via thrombolytics or thrombectomy) are another major factor driving disparities in outcomes among patients presenting to rural centers with ischemic stroke [14, 15]. Our study did not show a disparity among the groups for this essential time interval. One difference from our study, compared to other studies, would be the presence of the Critical Care Resuscitation Unit (CCRU).

The CCRU at UMMC was created in 2013 with the purpose of providing immediate critical care resuscitation for nontraumatic, time-sensitive medical conditions. For its operation, when our NCCU does not have a readily available bed, the CCRU would almost always accept patients with AIS-LVO for immediate transfer [16, 17]. Our ability to expedite transfers through the CCRU may have played a role in reducing disparities in time to recanalization [18]. Our current article in press at the journal of Critical Care Research and Practice (https://onlinelibrary.wiley.com/journal/3537) has suggested that although patients transferred to the CCRU from rural hospitals overall faced longer transport distances and times, they did not face meaningful differences in time to urgent interventions or hospital outcomes—though additional investigations are needed to examine this possibility [19]. Our study also identified no significant disparity in hospital complications among patients transferred from urban and rural centers for MT for AIS-LVO. A previous study by Grube et al. suggested that higher rates of hospital complications were associated with higher 90-day mortality among patients with ischemic stroke [20]. Thus, findings from this study may suggest that an effective system to assist and expedite the transfer of rural patients with AIS-LVO to a CSC, they can achieve similar outcomes as the urban patients.

### 4.1. Limitations

This study had several limitations. Our institution's development of a dedicated CCRU to expedite and facilitate critical care and emergent interventions for patients requiring transfer from outside hospitals is unique, and as such our findings may not be generalizable to other CSCs. Another limitation is the geography of the state this study was conducted in. Maryland has several geographical barriers to efficient ground travel in remote areas, such as mountains and bodies of water. The state healthcare system commonly uses air transport to minimize transport time, though our findings to date are inconclusive regarding the overall impact of air transport on outcomes [21]. In this study, we were unable to reliably identify the mode of transport (air vs. ground) for patients, and as such, were unable to assess whether these factors contributed to differences in functional neurologic outcome. This may have accounted for our finding that patients transferred from rural hospitals had similar LKW-recanalization time intervals despite facing a longer transport distance. Additionally, the state of Maryland is relatively small, and the overall ground distance from rural to urban hospital centers is inherently shorter compared to larger states. These logistical factors may also reduce the generalizability of this study.

The patients included in this study by necessity started their workup and care at outside institutions. As such, we have limited access to reliable data regarding some of their presenting data, such as their Alberta stroke program early CT (ASPECT) scores. Although our multivariable logistic regression considered most relevant demographic and clinical factors, the missing ASPECT score could have improved our model. Finally, we identified collinearity between two variables—occlusion of internal carotid vessels [VIF 8.4], and occlusion of more than one vessel [VIF 6.7]). However, removing these variables from the model reduced the AUROC and our models' goodness-of-fit (Hosmer–Lemeshow *p* values became < 0.05). Since they were relevant variables, we decided to keep them in the model nonetheless.

Finally, there may be variations across institutions in practice patterns regarding MT and post-thrombectomy care that are not reflected in our findings and may influence outcomes. In this study, we did not report the proportion of patients whose AIS evolved to large infarcts and were thus deemed ineligible for MT. Such factors should be controlled for in future investigations of this topic.

## 5. Conclusions

In this study involving patients with AIS-LVO who were transferred from either rural or urban areas to undergo MT at a Comprehensive Stroke Center, we identified no significant difference in the odds of having good neurological function at 90 days and no significant difference in time from LKW to successful recanalization via MT. Patients transferred from rural areas faced similar rates of complications during their hospital course. Further studies are necessary to confirm our observations.

## Figures and Tables

**Table 1:** Demographic information and clinical outcomes of patients with acute ischemic stroke from large vessel occlusion (AIS-LVO) and underwent mechanical thrombectomy.

Variables	All patients	Urban	Rural	Difference between groups	95% CI of difference	*p* value
	*N* = 526	*N* = 293	*N* = 233			
Age, mean (SD)	66 (14.4)	66 (15.0)	67 (13.7)	−0.73	−3.22, 1.76	0.56
Body mass index, mean (SD)	29 (4.3)	29 (4.5)	29 (4.1)	−0.2	−0.94, 0.55	0.61
Male, *N* (%)	269 (51)	146 (50)	123 (53)	−0.03	−0.11, 0.06	0.5
Ground distance of travel (km)	50.4 [17.0–73.0]	17.0 [13.0–28.0]	74.8 [64.0–94.4]	−57.8	−57.8, −49.0	0.001
Past medical history, *N* (%)						
Diabetes mellitus	97 (18)	65 (22)	32 (14)	0.08	0.02, 0.15	0.01
Hypertension	199 (38)	126 (43)	73 (31)	0.12	0.03, 0.2	0.01
Hyperlipidemia	134 (25)	83 (28)	51 (22)	0.06	−0.01, 0.14	0.09
Atrial fibrillation	98 (19)	63 (22)	35 (15)	0.07	−0.0, 0.13	0.06
Tobacco use	103 (20)	58 (20)	45 (19)	0.01	−0.06, 0.07	0.89
Any anticoagulation use, *N* (%)	55 (10)	38 (13)	17 (7)	0.06	0.0, 0.11	0.03
Admission laboratory values						
Sodium (mmol/L), mean (SD)	138 (2.6)	138 (2.7)	138 (2.5)	−0.09	−0.54, 0.37	0.72
Creatinine (mg/dL), mean (SD)	0.95 (0.57)	0.97 (0.68)	0.93 (0.41)	0.04	−0.06, 0.14	0.41
Glucose (mg/dL), median [IQR]	142 [117–142]	142 [113–142]	142 [127–142]	0	−8.19, 0.0	0.06
Platelet count (k/mcl), mean (SD)	230 (58.2)	227 (59.1)	233 (57.1)	−5.11	−15.16, 4.92	0.32
INR, median [IQR]	1.16 [1.10–1.16]	1.16 [1.0–1.16]	1.16 [1.1–1.16]	0	−0.06, 0.0	0.27
NIHSS, median [IQR]	17 [14–20]	17 [14–20]	17 [14–21]	−0.06	−1.06, 0.0	0.14
Occluded vessel, *N* (%)						
Internal carotid artery	98 (19)	44 (15)	54 (23)	−0.08	−0.15, −0.01	0.02
Middle cerebral artery	499 (95)	279 (95)	220 (94)	0.01	−0.03, 0.05	0.68
> 1 vessel	81 (15)	37 (13)	44 (19)	−0.06	−0.13, 0.0	0.05
Interventions						
IV thrombolysis, *N* (%)	160 (30)	101 (34)	59 (25)	0.09	0.01, 0.17	0.02
# Thrombectomy passes, median [IQR]	1 [1–2]	1 [1–2]	1 [1–2]	0	0.0, 0.0	0.1
TICI 2b/2c/3, *N* (%)	460 (87)	249 (85)	211 (91)	−0.06	−0.11, 0.0	0.06
Hospital course, *N* (%)						
Mechanical ventilation during hospitalization, *N* (%)	124 (0.24)	78 (0.27)	46 (0.2)	0.07	−0.0, 0.14	0.06
Tracheostomy, *N* (%)	25 (5)	13 (4)	12 (5)	−0.01	−0.05, 0.03	0.7
Pneumonia, *N* (%)	37 (7)	22 (8)	15 (6)	0.02	−0.04, 0.05	0.63
Hemorrhagic conversion, *N* (%)	40 (7.6)	26 (9)	14 (6)	0.03	−0.02, 0.07	0.25
Craniectomy, *N* (%)	13 (2)	9 (3)	4 (2)	0.01	−0.02, 0.04	0.32
HLOS (days), median [IQR]	7.3 [4.6–13.0]	7.9 [4.7–12.9]	6.2 [4.1–13.0]	1.64	−0.53, 3.38	0.21
90 day mRS score, *N* (%)						
90 day mRS 0–2	106 (20)	61 (21)	45 (19)	0.02	−0.05, 0.08	0.67

Abbreviations: HLOS, hospital length of stay; INR, international normalized ratio; IQR, interquartile; IV, intravenous; MCA, middle cerebral artery; mRS, modified Rankin score; NIHSS, National Institute of Health Stroke Score; TICI, thrombolysis in cerebral infarction.

**Table 2:** Predictors for good neurologic outcome for patients with acute ischemic stroke from large vessel occlusion (AIS-LVO) treated with mechanical thrombectomy, by multivariable logistic regression.

Variables	OR	95% CI	*p* value	VIF
Age	0.95	(0.93, 0.97)	0.001	1.49
NIHSS on arrival to CSC	0.92	(0.87, 0.96)	0.002	1.25
Interval from LWK-Recan	0.99	(0.99, 1.000)	0.045	1.59
History of hypertension	3.07	(1.3, 6.83)	0.006	2.5
History of atrial fibrillation	2.38	(1.13, 4.97)	0.021	1.75
Treatment with intravenous thrombolysis	1.93	(1.04, 3.58)	0.035	1.51
Symptomatic hemorrhagic conversion	0.27	(0.097, 0.75)	0.013	1.26
Occlusion of internal carotid artery	7.04	(1.09, 45.23)	0.04	8.39

*Note:* Hosmer–Lemeshow test: Df (8), Chi-square 8, *p* value = 0.39. Area under the receiver operating curve (AUROC): 0.84. LKW-Recan, time intervals from time of last known well to time achieving recanalization. Only statistically significant variables are presented here.

Abbreviations: CSC, Comprehensive Stroke Center; NIHSS, National Institute of Health Stroke Scale.

**Table 3:** List of all variables being used for the multivariable logistic regression.

Continuous variables	Categorical variables
Age	Rural
Body mass index	Gender-male
Distance of travel by ground	PMH diabetes mellitus
Serum sodium	PMH hypertension
Admission creatinine	PMH hyperlipidemia
Admission glucose	PMH atrial fibrillation
Admission platelet count	PMH tobacco use
Admission INR	Any anticoagulation at home
Admission NIHSS	Occlusion from the middle cerebral artery
Interval from last known well time to recanalization	Occlusion from more than one vessel
Number of thrombectomy passes	Occlusion of the internal carotid artery
	Intravenous thrombolytics
	TICI score
	Mechanical ventilation during hospitalization
	Symptomatic hemorrhagic conversion
	Tracheostomy
	Pneumonia
	Craniectomy

Abbreviations: INR, international normalized ratio; NIHSS, National Institute of Health Stroke Scale; PMH, past medical history; TICI, thrombolysis in cerebral infarction.

**Table 4:** Full results from the multivariable logistic regression.

Variables	OR	95% CI	*p* value	VIF
Age	0.95	(0.93, 0.97)	0.001	1.49
Gender-Male	0.95	(0.55, 1.64)	0.9	1.23
Body mass index	0.96	(0.91, 1.01)	0.12	1.25
Rural	0.96	(0.48, 1.92)	0.91	1.89
Distance of travel by ground	1.003	(0.99, 1.01)	0.43	1.8
Serum sodium	1.03	(0.94, 1.13)	0.54	1.17
Admission creatinine	0.62	(0.24, 1.55)	0.31	1.17
Admission glucose	0.99	(0.99, 1.00)	0.26	1.45
Admission platelet count	1.003	(0.99, 1.01)	0.21	1.24
Admission INR	0.73	(0.21, 2.53)	0.62	1.18
NIHSS on arrival to CSC	0.92	(0.88, 0.97)	0.002	1.25
Interval from LKW-Recan	0.99	(0.99, 1.00)	0.06	1.55
Number of thrombectomy passes	0.98	(0.75, 1.28)	0.89	1.3
PMH diabetes mellitus	1.24	(0.59, 2.61)	0.57	1.75
PMH hypertension	2.88	(1.31, 6.32)	0.01	2.45
PMH hyperlipidemia	1.6	(0.83, 3.11)	0.16	1.69
PMH atrial fibrillation	2.11	(1.02, 4.33)	0.04	1.7
PMH tobacco use	1.43	(0.77, 2.66)	0.26	1.32
Any anticoagulation at home	0.77	(0.33, 1.79)	0.55	1.41
Occlusion from the middle cerebral artery	1.24	(0.37, 4.14)	0.73	1.05
Intravenous thrombolytics	1.99	(1.09, 3.65)	0.03	1.48
TICI score	1.1	(0.39, 3.12)	0.86	1.47
Mechanical ventilation during hospitalization	1.08	(0.56, 2.08)	0.81	1.56
Symptomatic hemorrhagic conversion	0.32	(0.12, 0.86)	0.02	1.23
Tracheostomy	0.89	(0.27, 2.94)	0.85	1.38
Pneumonia	0.95	(0.38, 2.41)	0.92	1.29
Craniectomy	0.38	(0.07, 2.18)	0.28	1.2

*Note:* All variables were selected a priori and were entered into the model simultaneously.

Abbreviations: INR, international normalized ratio; LKW, last known well; NIHSS, National Institute of Health Stroke Scale; PMH, past medical history; TICI, thrombolysis in cerebral infarction.

## Data Availability

The data used to support the findings of this study are not available for the public due to IRB restrictions.
